# Inverse fluoxetine effects on inhibitory brain activation in non-comorbid boys with ADHD and with ASD

**DOI:** 10.1007/s00213-014-3837-2

**Published:** 2014-12-24

**Authors:** Kaylita Chantiluke, Nadia Barrett, Vincent Giampietro, Paramala Santosh, Michael Brammer, Andrew Simmons, Declan G. Murphy, Katya Rubia

**Affiliations:** 1 Department of Child and Adolescent Psychiatry/MRC Center for Social, Genetic and Developmental Psychiatry (SGDP), Institute of Psychiatry, King’s College London, 16 De Crespigny Park, PO46, London, SE5 8AF UK; 2 South London and Maudsley NHS Trust, London, UK; 3 Department of Neuroimaging, Institute of Psychiatry, King’s College London, London, UK; 4 NIHR Biomedical Research Centre for Mental Health at South London and Maudsley NHS Trust and Institute of Psychiatry, King’s College London, London, UK; 5 Department of Forensic and Developmental Sciences & the Sackler Institute for Translational Neurodevelopment, Institute of Psychiatry, King’s College London, London, UK

**Keywords:** ADHD, ASD, Motor response inhibition, Stop task, fMRI, Serotonin, Fluoxetine

## Abstract

**Rationale:**

Attention deficit hyperactivity disorder (ADHD) and autism spectrum disorder (ASD) are often comorbid and have both performance and brain dysfunctions during motor response inhibition. Serotonin agonists modulate motor response inhibition and have shown positive behavioural effects in both disorders.

**Aims:**

We therefore used functional magnetic resonance imaging (fMRI) to investigate the so far unknown shared and disorder-specific inhibitory brain dysfunctions in these two disorders, as well as the effects of a single dose of the selective serotonin reuptake inhibitor fluoxetine.

**Methods:**

Age-matched boys with ADHD (18), ASD (19) and healthy controls (25) were compared with fMRI during a stop task measuring motor inhibition. Patients were scanned twice, under either an acute dose of fluoxetine or placebo in a double-blind, placebo-controlled randomised design. Repeated measures analyses within patients assessed drug effects. To test for potential normalisation effects of brain dysfunctions, patients under each drug condition were compared to controls.

**Results:**

Under placebo, relative to controls, ASD boys showed overactivation in left and right inferior frontal cortex (IFC), while ADHD boys showed disorder-specific underactivation in orbitofrontal cortex (OFC) and basal ganglia. Under fluoxetine, the prefrontal dysfunctions were no longer observed, due to inverse effects of fluoxetine on these activations: fluoxetine downregulated IFC and OFC activation in ASD but upregulated them in ADHD.

**Conclusions:**

The findings show that fluoxetine normalises frontal lobe dysfunctions in both disorders via inverse effects, downregulating abnormally increased frontal activation in ASD and upregulating abnormally decreased frontal activation in ADHD, potentially reflecting inverse baseline serotonin levels in both disorders.

**Electronic supplementary material:**

The online version of this article (doi:10.1007/s00213-014-3837-2) contains supplementary material, which is available to authorized users.

## Introduction

Attention deficit hyperactivity disorder (ADHD) is a neurodevelopmental disorder defined by age-inappropriate levels of inattention, impulsivity and hyperactivity (American Psychiatric Association 2000). Autism spectrum disorder (ASD) is defined by impairments in communication, social interaction and by restricted and repetitive behaviours (American Psychiatric Association 2000). There is increasing evidence for comorbidity between disorders (Rommelse et al. 2011; Simonoff et al. 2008; van der Meer et al. 2012) and shared executive function deficits (Corbett et al. 2009; Geurts et al. 2004), in particular in motor response inhibition (Alderson et al. 2007; Corbett et al. 2009; Lipsyzc and Schachar 2010; Robinson et al. 2009), albeit less consistently in ASD (Ozonoff and Strayer 1997; Raymaekers et al. 2007). Furthermore, this has been associated with impulsiveness in ADHD and motor stereotypies in ASD (Langen et al. 2011). This overlap was highlighted by recent changes to the DSM-V that allows co-diagnosis of ADHD and ASD (http://www.dsm5.org/).

In ADHD, there is consistent evidence of underactivation compared to controls in inferior/orbitofrontal frontal cortex (IFC/OFC), supplementary motor area (SMA) and caudate/thalamus during inhibition tasks (Cubillo et al. 2012; Rubia 2011; Rubia et al. 2014; Rubia et al. 1999; Rubia et al. 2005b; Cortese et al. 2012; Hart et al. 2013). In children with ASD, however, no study has investigated the neurofunctional underpinnings of response inhibition. In adults with ASD, fMRI studies report inconsistent findings of increased activation in left IFC and decreased activation in right IFC and anterior cingulate cortex (ACC) during the go/no-go task (Kana et al. 2007; Schmitz et al. 2006). Therefore, a key question is whether the underlying neurobiology of shared cognitive phenotypes is shared or disorder-specific.

Serotonin (SE) is involved in impulsiveness and motor inhibition (Robbins et al. 2010) and although selective serotonin reuptake inhibitors (SSRIs) such as citalopram had no effect on SSRT in healthy subjects (Chamberlain et al. 2006), fMRI studies showed that SSRIs and acute tryptophan depletion (ATD), which enhance/reduce SE levels, enhance/decrease inhibitory IFC/OFC-striatal activation, respectively (Lamar et al. 2012; Lamar et al. 2009; Rubia et al. 2005a).

Furthermore, there is evidence that 5-HT is involved in the pathology of both ADHD and ASD. Thus, polymorphisms of serotonergic genes have been associated with both ADHD and ASD (Rommelse et al. 2010; Sinzig and Lehmkuhl 2007). Moreover, biochemical serotonergic abnormalities have been implicated in both ADHD (Oades 2007) and ASD (Zafeiriou et al. 2009). Despite evidence for SE abnormalities in both disorders, few studies have investigated the clinical efficacy of SSRIs in ADHD and ASD. The SSRI fluoxetine, for example, has been shown to improve inattentiveness and hyperactivity in comorbid children with ADHD (Barrickman et al. 1991; Gammon and Brown 1993; Quintana et al. 2007) and to improve communication, social interaction and stereotyped behaviours in children with ASD (DeLong et al. 2002; DeLong et al. 1998; Hollander et al. 2005), albeit citalopram was not effective (King et al. 2009).

Given the evidence for abnormalities in SE (Oades 2007; Zafeiriou et al. 2009) and inhibition in both disorders (Alderson et al. 2007; Corbett et al. 2009; Geurts et al. 2004; Robinson et al. 2009; Rommelse et al. 2011) and serotonergic mediation of inhibitory control (Anderson et al. 2008; Lamar et al. 2012; Lamar et al. 2009; Robbins et al. 2010; Rubia et al. 2005a), an important question that may elucidate the potential neurotransmitter underpinnings of these cognitive abnormalities is whether SE modulates the inhibitory network in *both* ADHD and ASD and whether this modulation differs between disorders.

The aim of this fMRI study was therefore to investigate (1) shared and disorder-specific brain dysfunctions in ADHD and ASD boys during a tracking stop task and (2) shared and disorder-specific neurofunctional effects of an acute dose of fluoxetine on these inhibitory (dys)functions in both disorders. It has been argued that inferior frontal activation during stop task performance may be confounded by the attentional oddball effect of the low frequency appearance of stop signals (Hampshire et al. 2010; Pauls et al. 2012a). Given that in this study we were particularly interested in the effects of fluoxetine on inhibitory rather than on attention networks, in our fMRI analysis we contrasted successful stop trials with failed stop trials in order to control for the attentional oddball effect of the low frequency appearance of the stop trials.

We hypothesised that, under placebo, ADHD boys compared to controls would show decreased IFC and caudate activation (Cortese et al. 2012; Cubillo et al. 2012; Cubillo et al. 2014; Hart et al. 2013; Rubia 2011; Rubia et al. 1999; Rubia et al. 2005b), whereas ASD boys would show increased left frontal activation (Schmitz et al. 2006). We further hypothesised that fluoxetine compared to placebo, given its beneficial effects on symptoms, would normalise the abnormal activations in each disorder, i.e. it would increase the reduced fronto-striatal activation in ADHD, but reduce the increased left frontal abnormalities in ASD.

## Materials and methods

### Participants

Sixty-two right-handed boys (Edinburgh Handedness Inventory) (Oldfield 1971) (25 controls, 18 with ADHD and 19 with ASD) aged 10–17, IQ > 70 (Wechsler Abbreviated Scale of Intelligence-Revised (WASI-R) (Wechsler 1999) participated in this study.

ADHD boys had a clinical diagnosis of non-comorbid ADHD, inattentive/hyperactive-impulsive combined subtype, as assessed by an experienced child psychiatrist using the standardised Maudsley diagnostic interview that assesses ADHD according to DSM-IV criteria (Goldberg and Murray 2006). They scored above clinical threshold for ADHD symptoms on the Strengths and Difficulties Questionnaire (SDQ) (Goodman and Scott 1999) and the Conners’ Parent Rating Scale-Revised (CPRS-R) (Conners et al. 1998). Four of the ADHD boys were medication-naïve, three had ceased taking methylphenidate for a year (1), or 3 months (2) and 11 received chronic stimulants, but had a 48-h medication washout prior to scanning. ADHD boys were excluded if they scored above 15 on the Social Communication Questionnaire (SCQ) (Rutter et al. 2003).

ASD diagnosis was made using ICD-10 (World Health Organisation (WHO) 1994) diagnostic criteria and confirmed by the Autism Diagnostic Interview-Revised (ADI–R) (Lord et al. 1994) and the Autism Diagnostic Observation Schedule (ADOS) (Lord et al. 2000). All ASD subjects were medication-naïve apart from one patient, who took melatonin (but underwent a 2-week medication washout). ASD exclusion criteria included a score above 7 on the hyperactivity/inattention subscale of the SDQ or a *t* score above 70 on the DSM-IV subscale of the CPRS-R.

Exclusion criteria for all were comorbidity with other psychiatric or neurological disorders and drug/alcohol dependency. Patients were recruited from local clinics and support groups.

Patients were scanned twice in a double-blind, randomised, placebo-controlled design, using a Latin square randomization design for counter-balanced effects. Due to the half-life of fluoxetine (1–3 days) and its metabolite norfluoxetine (5–16 days) (Wong et al. 1995), each scan was 3–4 weeks apart. To ensure that fluoxetine had reached its peak plasma levels, after 5–8 h (Wong et al. 1995), patients were scanned 5 h after administration. Liquid fluoxetine was titrated to age and weight (see supplement). Placebo was equivalent amounts of peppermint water with similar taste to fluoxetine.

Twenty-five healthy, handedness and age-matched boys were recruited by advertisement and scored below clinical thresholds on the SDQ, SCQ and CPRS.

Participants gave written informed consent/assent. The study was approved by the local Ethics Committee. Participants were paid £50 for each scan.

For recruitment, demographic and clinical details of participants, see Table 1.

**Table 1:** Sample characteristics for healthy control boys and patients with ADHD and ASD

Variables	Controls (25) mean (SD)	ADHD (18) mean (SD)	ASD (19) mean (SD)
Age (years)	13.4 (2.4)	14.3 (1.8)	14.7 (2.0)
IQ	109 (13)	95 (11)	112 (15)
SDQ hyperactive-impulsive/inattentive subscale	1.8 (1.6)	9.2 (0.9)	4.5 (1.8)
SDQ—emotional distress subscale	0.5 (0.8)	3.6 (3.0)	4.2 (3.0)
SDQ—conduct subscale	0.3 (0.7)	5.0 (2.4)	2.1 (2.0)
SDQ—peer relations subscale	.6 (1.1)	3.4 (2.5)	6.1 (2.4)
SDQ—prosocial behaviour subscale	9.1 (1.3)	6.7 (2.3)	5.1 (2.3)
SDQ—total scores	3.3 (2.9)	21.2 (4.9)	16.8 (5.7)
SCQ total	1.6 (2.7)	7.0 (3.4)	23.5 (5.5)
CPRS-R total *T* score	44 (3)	83 (7)	57 (8)
ADOS communication scores	–	–	2 (1)
ADOS social interaction scores	–	–	7 (4)
ADOS communication and social scores	–	–	9 (5)
ADOS stereotyped behaviour scores	–	–	1 (1)
ADI communication scores	–	–	14 (4)
ADI social Interaction scores	–	–	17 (5)
ADI stereotyped scores	–	–	6 (3)

*SDQ* Strengths and Difficulties Questionnaire, *SCQ* Social Communication Questionnaire, *CPRS-R* Conners’ Parent Rating Scale-Revised, *ADOS* Autism Diagnostic Observation Schedule, *ADI* Autism Diagnostic Interview

### FMRI paradigm: stop task

Subjects practised the task once prior to each scan under supervision of the researcher who made sure the participants understood the task and performed accordingly (by inhibiting more or less 50 % of trials). The 8 min 49 s minute visual tracking stop task requires withholding of an already triggered motor response to a go stimulus when it is followed unpredictably by a stop signal (Cubillo et al. 2014; Rubia et al. 2011; Rubia et al. 2003; Rubia et al. 2005b; Rubia et al. 2007). Subjects have to respond as quickly as possible to left or right pointing “go” arrows (500 ms duration, 80 % of 294 trials) with a left or right (thumb) button press, followed by a gap of 1100 to 1500 ms (which makes up a mean trial length of 1.8 s; jittered between 1.6 and 2 s to optimise statistical efficiency). In 20 % of trials (60), pseudo-randomly interspersed, and at least 3 repetition times apart for adequate separation of the hemodynamic response, go signals are followed (about 250 ms later) by arrows pointing upwards (300 ms duration) (stop signals), and subjects have to inhibit their motor response. A tracking algorithm changes the time interval between go and stop-signal onsets according to each subject’s inhibitory performance, which is recalculated after each stop signal based on the average percentage of inhibition over previous stop trials to provide 50 % successful and 50 % unsuccessful inhibition trials (Supplementary Figure S1).

Given that the contrast of stop-go trials is confounded by the attentional oddball effect of the low frequency of stop trials (20 %) relative to go trials (80 %), which also activates IFC (Hampshire et al. 2010; Pauls et al. 2012b), for the fMRI analysis, brain activation to the 50 % unsuccessful stop trials was subtracted from the 50 % successful stop trials, controlling for the attentional oddball effect of the infrequent stop signal appearance.

The dependent inhibition variable is the stop signal reaction time (SSRT), calculated by subtracting the mean stop signal delay (SSD: the average time between go and stop signal at which the subject managed to inhibit to 50 % of trials) from the mean reaction time (MRT) to go trials, i.e. MRT-SSD (Logan et al. 1997). MRT, intrasubject standard deviation of MRT, and omission error percentage are variables of the executive process of the task.

## Data analysis

### Analysis of performance data

MANOVAs compared performance variables between controls and the two patient groups under placebo and between controls and the two patient groups under fluoxetine. Multiple repeated-measures ANOVAs within the two patient groups with group (ADHD, ASD) as independent factor and drug condition (placebo/fluoxetine) as repeated measures were conducted to test for group by medication interaction effects on performance.

### FMRI image acquisition

Gradient-echo echoplanar MR imaging (EPI) data were acquired on a General Electric Signa 3T Horizon HDx system at the Centre for Neuroimaging Sciences, Institute of Psychiatry, King’s College London, UK. A semiautomated quality control procedure ensured consistent image quality. A quadrature birdcage headcoil was used for RF transmission and reception. In each of 28 non-contiguous planes parallel to the anterior-posterior commissure, 296 T2*-weighted MR images depicting BOLD contrast covering the whole brain were acquired with TE = 30 ms, TR = 1.8 s, flip angle = 75°, in-plane voxel-size = 3 mm, slice thickness = 5.5 mm and slice-skip = 0.5 mm. A whole-brain high-resolution structural scan (inversion recovery gradient echo planar image) on which to superimpose the individual activation maps, was also acquired in the inter-commissural plane with TE = 30 ms, TR = 3 s, flip angle = 90°, 43 slices, slice thickness = 3.0 mm, slice skip = 0.3 mm and in-plane voxel-size = 1.875 mm.

All subjects performed three other fMRI tasks which are not reported here.

### FMRI image analysis

The XBAM software package was used (http://www.brainmap.co.uk/) (Brammer et al. 1997) which makes no normality assumptions (often violated in fMRI data), but instead uses median statistics to control outlier effects and permutation rather than normal theory-based inference (Thirion et al. 2007).

Individual- and group-level analyses are described in further detail elsewhere (Brammer et al. 1997; Cubillo et al. 2014; Rubia et al. 2003; Rubia et al. 2005b; Rubia et al. 2007) and in the supplementary material. Briefly, the fMRI data were realigned to minimise motion-related artefacts and smoothed using a Gaussian filter (FWHM 8.82 mm) (Bullmore et al. 1999a). Further data analysis included slice timing correction and the residual effects of motion were regressed out from the time series (using the estimated motion parameters) before fitting a general linear model. Time-series analysis of individual subject activation was performed with a wavelet-based resampling method previously described (Bullmore et al. 2001). We convolved the task epoch of the contrasts of interest (i.e. successful stop—implicit go trials; failed stop trials—implicit go trials) and the higher level contrast (successful stop-go trials minus unsuccessful stop-go trials) with two Poisson model functions (delays of 4 and 8 s). Individual activation maps were recalculated by testing the goodness-of-fit of this convolution with the BOLD time series; the goodness-of-fit calculation used the ratio of the sum of squares of deviations from the mean intensity value due to the model (fitted time series) divided by the sum of squares due to the residuals (original time series minus model time series). This statistic, the sum of squares (SSQ) ratio, was used in further analyses. Using rigid body and affine transformation, the individual maps were registered into Talairach standard space. A group brain activation map was then produced for each medication condition and each experimental condition (see supplementary material).

### ANCOVA between-group difference analyses

For between-group comparisons between controls and patients under either placebo or fluoxetine, one-way ANCOVA analyses with group as factor and rotational and translation movement in Euclidian 3-D space as covariate, were conducted using randomization-based tests for voxel or cluster-wise differences as described in detail elsewhere (Bullmore et al. 2001; Bullmore et al. 1999b). For these between-group comparisons, less than one false activated cluster was expected at *p* < 0.05 for voxel and *p* < 0.01 for cluster comparisons. Given that right IFC was an a priori hypothesised region, we used a more lenient cluster level threshold of *p* < 0.05 and also conducted a region of interest analysis using the IFC as a mask. Then the standardised BOLD response values for each participant were extracted for the significant clusters of the ANCOVA analyses and post hoc *t*-tests (correcting for multiple comparisons using least significant difference (LSD)) were conducted to identify the direction of the group differences.

### ANCOVA within-patient interaction effects

A 2 × 2 ANCOVA (2 medication conditions, 2 groups) with rotational and translation movement as covariate was conducted using randomised-based testing for voxel or cluster-wise differences as described elsewhere (Bullmore et al. 2001). Less than one false positive activation cluster was expected at *p* < 0.05 at voxel and *p* < 0.01 at cluster level. For our a priori hypothesised region in right IFC, a more lenient *p* < 0.05 was used as well as a region of interest analysis using the IFC as a mask. Statistical measures of BOLD response for each participant were then extracted in each of the significant clusters and post hoc *t* tests (corrected for multiple comparisons with LSD) were conducted to identify the direction of the interaction effects.

## Results

### Participant characteristics

ANOVAs showed no significant group differences in age (*F* (df = 2,61) = 3; n.s.) but in IQ (*F* (df = 2,61) = 10, *p* < 0.001) which was significantly lower in ADHD relative to control and ASD boys (*p* < 0.005), who did not differ. Given that ADHD children have typically lower IQ than their healthy peers (Bridgett and Walker 2006), data were not covaried for IQ, as covarying for a measure that is associated with the condition would violate ANCOVA assumptions (Dennis et al. 2009). Nonetheless, to assess the potential impact of IQ, analyses were repeated with IQ as covariate. For group differences in clinical measures, see Table 1.

### Performance data

As expected, all subjects achieved approximately 50 % probability of inhibition, suggesting the algorithm was successful and there were no differences between controls and patient groups in this measure for either placebo (*F* (df = 2,61) = 1.2; *p* = n.s.) nor fluoxetine (*F* (df = 2,61) = 2.7, *p* = n.s.) nor were there any group or group by medication interaction effects for the within-patients analysis (F (df = 2,61) < 1, *p* = n.s.). MANOVA showed a trend for a significant group effect for placebo (*F* (df = 8,114); *p* < 0.08), which was due to increased omission errors between controls and patients under placebo (*F* (df = 2,61) = 4, *p* < 0.02). Post hoc analyses (corrected for multiple testing using least significance difference (LDS)) showed that this was due to ADHD boys having increased omission errors than both control (*p* < 0.05) and ASD boys (*p* < 0.01) (Table 2). MANOVA for fluoxetine showed no significant group effect (*F* (df = 2,61) = 1.2, *p* = n.s.).

**Table 2:** Performance measures for the stop task for healthy controls, ADHD and ASD groups

Performance measure	Controls		ADHD placebo		ADHD fluoxetine		ASD placebo		ASD fluoxetine	
	Mean	SD	Mean	SD	Mean	SD	Mean	SD	Mean	SD
SSRT	161	107	132	81	142	109	140	125	142	103
PI	51	3	50	3	49	4	50	3	49	4
SSD	462	23	497	27	490	30	479	27	480	29
MRT to go trials	623	104	629	86	632	109	618	102	622	113
SD for MRT to go trials	174	39	194	44	194	60	168	39	166	43
Omission errors^a^	5	5	9	8	9	9	3	5	3	3

*PI* percentage inhibition, *SSRT* stop signal reaction time, *SSD* average stop signal delay, *MRT* mean reaction time

SD = intrasubject variability of reaction time (in ms)

^a^ADHD > C, ASD

Within the patient groups there were no significant group (df = 1,35) or group by medication interaction effects (df = 1,35) for any variables (*F* < 1 for all measures).

Given that ADHD patients performed the task twice, but controls only once, we also measured practice effects in the ADHD group using repeated measures *t* tests between performance measures at scans 1 and 2. No significant differences were found (*F* (df = 1,17) = 0.02, *p* = n.s.).

### FMRI data

#### Movement

The Euclidean formula described below was used to produce a 3-D movement value known as *d* which is representative of movement in *x*, *y* and *z*. The formula was used to produce a *d* value for the maximum translation and maximum rotation of each participant.

$$ d=\sqrt{{\left({p}_1-{q}_1\right)}^2+{\left({p}_2-{q}_2\right)}^2+{\left({p}_3-{q}_3\right)}^2.} $$

Repeated measures ANOVAs showed no significant group by movement interaction effects in the Euclidean measures of maximum xyz rotation (*F* (df = 4118) = 2, *p* = 0.14) or maximum xyz translation (*F*(df = 4118) = 1, *p* = 0.58). Nevertheless, to eliminate any potential effects of non-significant variance in motion, these Euclidean motion parameters for maximum xyz translation and rotation were used as covariates in the fMRI analyses.

Within-group activations are reported in the supplementary results section and in supplementary Figs. S2 and S3.

### Between-group differences between controls and patients under placebo

**Fig. 1:**
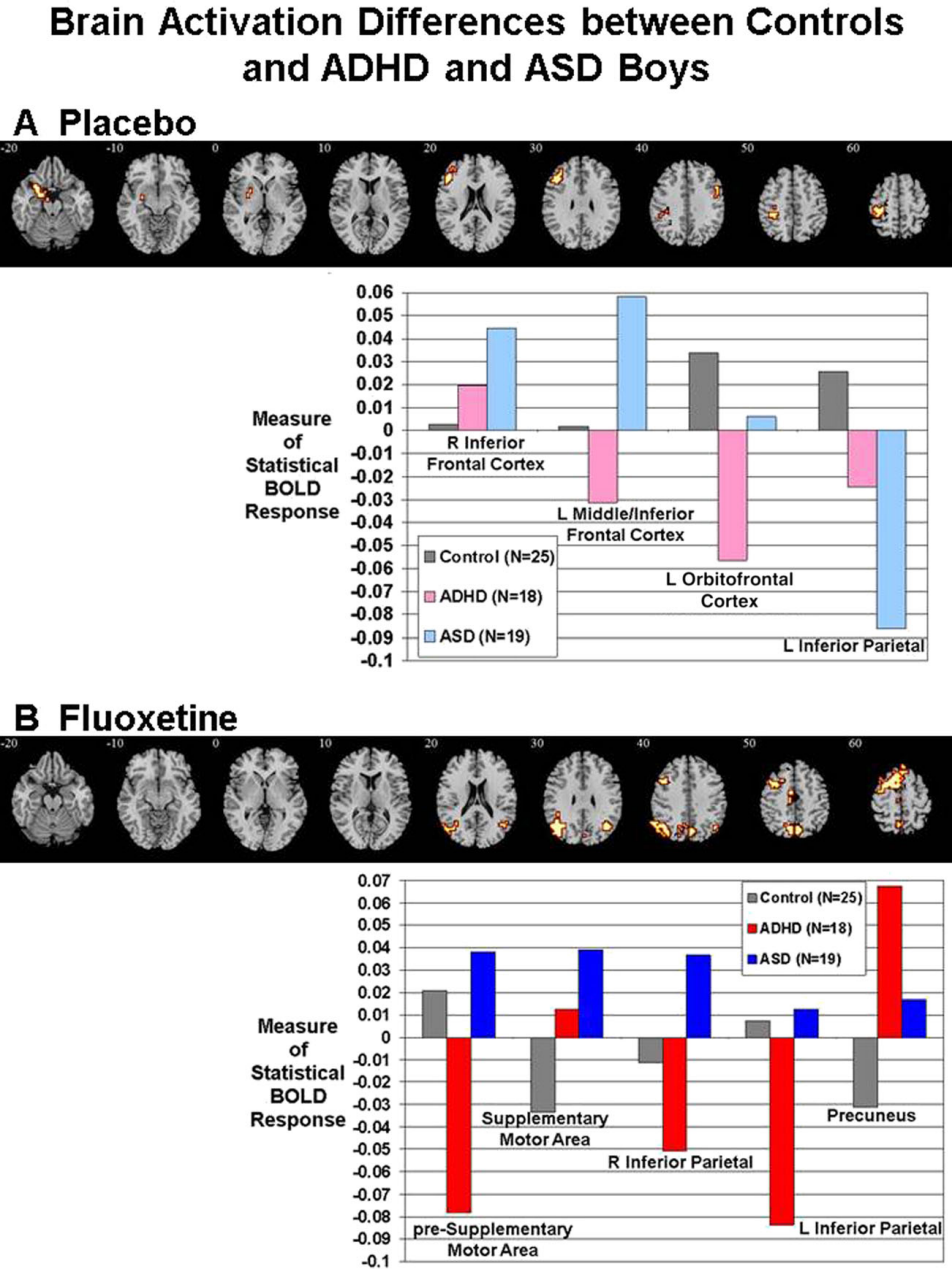
Between-group brain activation differences between controls and patients under placebo or fluoxetine for the contrast of successful stop with unsuccessful stop trials. **a** Axial sections showing the between-group ANCOVA comparison findings between controls and patients under placebo. Shown underneath are the statistical measures of BOLD response for each of the three groups for each of the brain regions that showed a significant group effect. **b** Axial sections for the between-group ANCOVA comparison between controls and patients under fluoxetine. Talairach *z* coordinates are indicated for slice distance (in mm) from the intercommissural line. The right side of the image corresponds to the right side of the brain

ANCOVA between controls and patients on placebo showed significant group differences in left middle/IFC, left OFC/superior temporal lobe (STL) reaching into putamen and globus pallidus and in left inferior parietal lobe (IPL). The hypothesised difference in right IFC was observed at a more lenient *p* < 0.05 (Fig. 1a, Table 3) and confirmed by a region of interest analysis (Talairach coordinates (x;y;z) = 54;7;26; BA 45; 10 voxels).

**Table 3:** Brain activation differences between controls and patients on placebo or fluoxetine

Post hoc group differences	Brain regions of activation differences	Brodmann area (BA)	Peak Talairach coordinates (x;y;z)	Voxels	Cluster *p* value
Placebo					
C < ASD	R inferior frontal cortex	44	54;15;26	16	0.03
C, ADHD < ASD	L inferior/middle frontal cortex	44/45/46/9	−40;30;23	82	0.002
C, ASD > ADHD	L STL/OFC/putamen/globus pallidus	38/47/11/25	−29;11;−26	73	0.008
C > ADHD > ASD	L inferior parietal lobe	40	−29;−22;40	97	0.002
Fluoxetine					
C, ASD > ADHD	L + R pre-SMA/premotor/superior frontal cortex	6/8	−11;4;63	203	0.0006
C < ADHD, ASD	SMA proper	6/8/9	−4;−11;50	42	0.007
ADHD < C < ASD	R inferior parietal lobe/angular gyrus	6	36;−63;30	50	0.004
C, ASD > ADHD	L inferior parietal lobe/middle temporal	40/39	−47;−63;26	188	0.0002
C < ASD < ADHD	Precuneus	40/39	7;−67;43	114	0.002

*STL* superior temporal lobe, *OFC* Orbitofrontal cortex, *SMA* supplementary motor area

Post hoc analyses showed that the group effect in right IFC was due to significantly increased activation in ASD relative to control boys (*p* < 0.005) and trend-wise compared to ADHD boys (*p* < 0.08). In left middle/IFC, ASD patients had significantly increased activation compared to both control and ADHD boys (*p* < 0.005) who activated this region during failed inhibition and had a trend-wise reduction relative to controls (*p* < 0.08). In left OFC/STL/basal ganglia both control and ASD boys, who did not differ from each other, showed increased activation relative to ADHD boys who activated this area during failed inhibition (*p* < 0.005). Left IPL was significantly reduced in both patient groups relative to controls (*p* < 0.05), with ASD patients activating this area significantly more during failed inhibition than ADHD (*p* < 0.05).

To test whether group effects were related to task performance, we correlated all clusters with SSRT and omission errors, since they were lower in ADHD relative to controls and ASD. The (reduced) activation in left OFC/basal ganglia in ADHD was significantly negatively correlated with omission errors (*r* = −0.5, *p* < 0.05). No other correlations were significant.

### Between-group differences between controls and patients under fluoxetine

There were significant group differences in bilateral pre-SMA/superior frontal cortex, SMA proper, left IPL/middle temporal lobe, right IPL and precuneus (Fig. 1b, Table 3). Post hoc analyses showed that control and ASD boys, who did not differ, activated pre-SMA/superior frontal cortex significantly more during successful inhibition than ADHD boys who activated this cluster more during failed inhibition (*p* < 0.0001). SMA proper was significantly more activated in patients, who did not differ relative to controls, who activated this region more during failed inhibition (*p* < 0.05). In bilateral IPL, controls and ASD boys had increased activation during successful inhibition relative to ADHD boys who activated this region more during failed inhibition (*p* < 0.05). In right IPL, ASD boys also had increased activation relative to controls (*p* < 0.05). In precuneus, ADHD boys showed significantly enhanced activation for successful inhibition relative to both ASD (*p* < 0.05) and controls who activated this region during failed inhibition and differed relative to ASD (*p* < 0.05). Correlations with behaviour showed that activation in pre-SMA/superior frontal cortex during successful inhibition was negatively correlated with SSRT in the ASD group (*r* = −0.532, *p* < 0.05). Activation in precuneus was negatively correlated with SSRT in the ADHD group (*r* = −0.493, *p* < 0.05).

### Effect size comparisons of case-control conditions to test for significant “normalisation” effects

To establish whether the group differences between controls and patients under each drug condition (ADHD/ASD under placebo versus controls; ADHD/ASD under fluoxetine versus controls) were significantly different, we directly compared the effect sizes (ES) of the group differences from the two case-control comparisons (Matthews and Altman 1996).

When comparing two effect sizes, the *z* test can evaluate the likelihood of whether they are significantly heterogeneous. The difference between the two effect sizes can be considered a normalized variable, where the standard error of the difference is a combination of the standard errors of the two comparisons. Based upon this, the probability of a type I error can be calculated using the formula: *p* (*α*) = (es_1_ − es_2_)/sqrt(se_1_^2^ + se_2_^2^).

For the abnormalities in the ADHD patients, the effect sizes of the ADHD placebo-control contrast was not significantly different from the effect sizes of the ADHD fluoxetine-control comparison for left OFC (*z* = −0.9, *p* = n.s.), right IFC (*z* = −0.6; *p* = n.s.) or the left inferior parietal activation clusters (*z* = 0.53, *p* = n.s.), suggesting that the normalisation effects in these clusters were not statistically significant.

For the abnormalities in the ASD patients, however, the effect sizes for the ASD placebo-control contrast were significantly different from the effect sizes for the ASD fluoxetine-control contrast for left IFC (*z* = 2.8, *p* < 0.005) and right IFC (*z* = −2.5, *p* < 0.01), both of which were enhanced under placebo but normalised under fluoxetine, and for left inferior parietal lobe, which was significantly reduced in ASD relative to controls under placebo, but enhanced under fluoxetine (*z* = 2.3, *p* < 0.02). The findings show that all normalisation effects in these clusters were statistically significant.

### Control for IQ

To assess the potential impact of IQ on case-control group differences, all analyses were repeated with IQ as a covariate. All clusters remained at a *p* < 0.01, apart from left OFC in the placebo between-group ANCOVA and right IPL in the fluoxetine between-group ANCOVA, which survived at a more lenient *p* < 0.05.

### Within-patients group by medication interaction effects

**Fig. 2:**
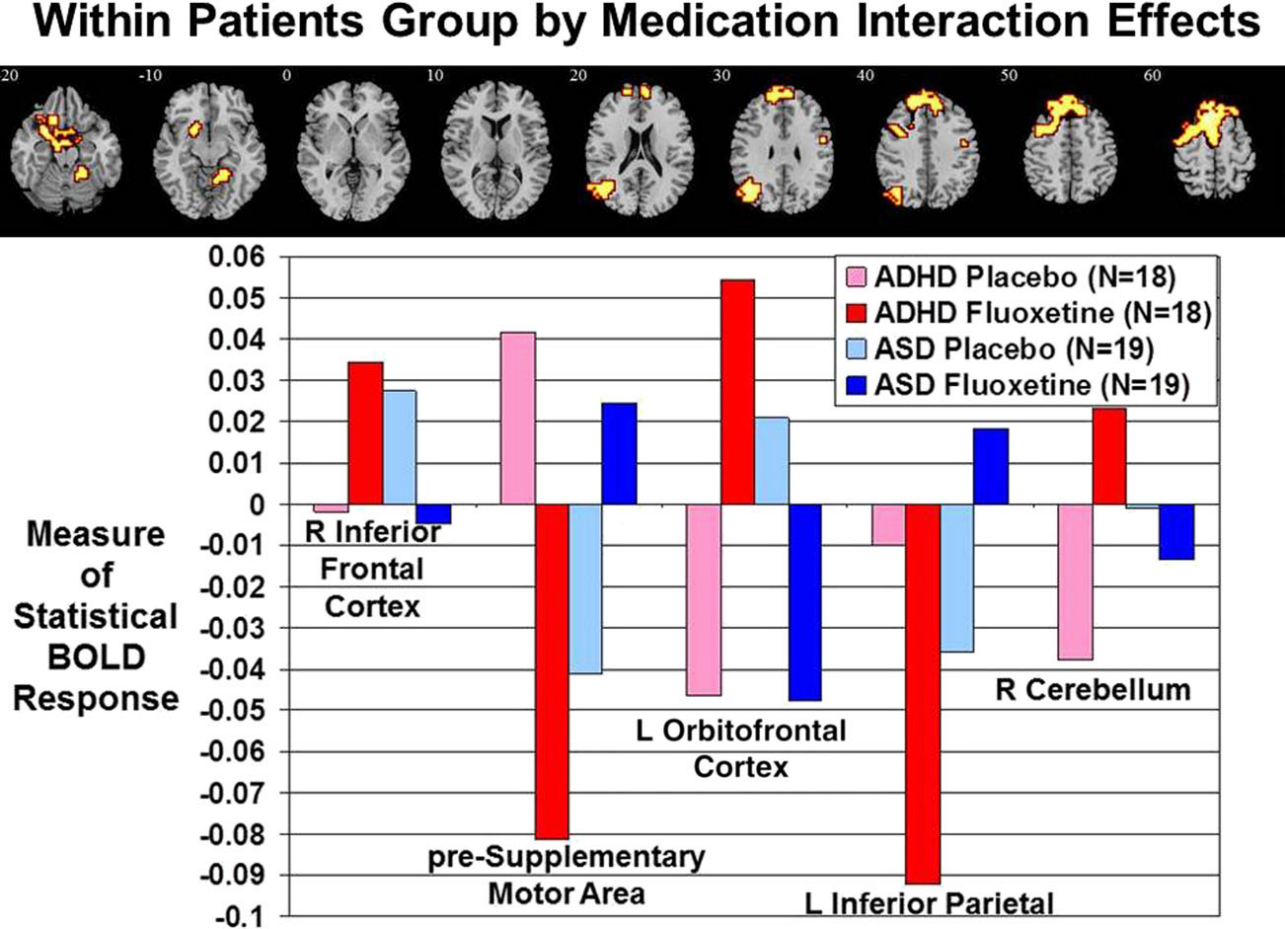
Within-patient group by medication interaction effects. Axial sections showing within-patient group by medication interaction effects for the contrast of successful stop-unsuccessful stop trials. Shown underneath are the statistical measures of BOLD response for each of the brain regions that showed a significant group by medication interaction effect within patients. Talairach *z* coordinates are indicated for slice distance (in mm) from the intercommissural line. The right side of the image corresponds to the right side of the brain

ANCOVA analysis with group as dependent variable and drug as within-group variable showed a significant group by medication interaction effect in five clusters. (1) Fluoxetine reduced pre-SMA activation in ADHD boys, whereas it increased it in ASD boys during successful inhibition (*p* < 0.005). (2) Fluoxetine increased activation in left OFC/STL/putamen/globus pallidus in ADHD during successful inhibition relative to placebo, but reduced it in ASD (*p* < 0.05). (3) Both patient groups activated left IPL during failed inhibition under placebo, but ADHD boys activated this region more during failed inhibition and ASD boys more during successful inhibition under fluoxetine (*p* < 0.0001). (4) Right cerebellum activation was increased under fluoxetine in ADHD relative to placebo for successful inhibition, while ASD boys activated this area during failed inhibition under both placebo and fluoxetine (*p* < 0.05). (5) At a more lenient *p* < 0.05, relative to placebo, fluoxetine increased activation in the hypothesised right IFC during successful inhibition in ADHD and decreased it in ASD (*p* < 0.05) (Fig. 2, Table 4). The same cluster was also confirmed by a region-of-interest analysis (Talairach coordinates (x;y;z) = 51;4;30; BA 45; 7 voxels).

**Table 4:** Group by drug interaction effects within ADHD and ASD patients

Brain regions of activation	Brodmann area (BA)	Peak Talairach coordinates (x;y;z)	Voxels	Cluster *p* value
R inferior/middle frontal cortex	44/9	51;0;26	21	0.03
R pre-SMA/premotor/superior frontal cortex	6/8	−7:33;56	1085	0.0003
L STL/OFC/putamen/globus pallidus	38/47/11/25	−4;−7;−26	452	0.004
L inferior parietal/middle/STL/occipital lobe	39/40/22/19	−43;−67;30	237	0.008
R cerebellum		22;−41;−17	139	0.0005

*STL* superior temporal lobe, *OFC* orbitofrontal cortex, *SMA* supplementary motor area

No significant correlations with performance were observed. All findings remained when IQ was covaried for.

## Discussion

The findings reveal opposite brain dysfunction patterns in inhibitory brain regions in ADHD and ASD as well as opposite disorder-dissociated modulation effects of fluoxetine on these neurofunctional abnormalities. Relative to controls, ADHD patients showed reduced left OFC/basal ganglia activation, whereas ASD patients showed enhanced bilateral ventrolateral prefrontal activation relative to ADHD and controls. Fluoxetine normalized these disorder-dissociated brain abnormalities relative to controls, via disorder-dissociated opposite effects within patients: fluoxetine enhanced the abnormally reduced OFC/basal ganglia activation in ADHD, but reduced it in ASD. In contrast, it reduced the abnormally enhanced IFC activation in ASD, but enhanced it in ADHD. However, rigorous testing for the significance of these normalisation effects showed that these were only significant for the abnormality clusters in ASD. Furthermore, fluoxetine also had opposite disorder-dissociated effects on other inhibition areas such as pre-SMA and cerebellum: these were, respectively, enhanced (SMA) and reduced (cerebellum) in ADHD with fluoxetine relative to placebo, but reduced (SMA) and enhanced (cerebellum) in ASD.

The finding of disorder-specific increased activation in ASD relative to controls and ADHD in the left and right (trend-wise significant relative to ADHD) IFC extends our previous finding of increased left IFC activation in young adults with Asperger's syndrome during a go/no-go task (Schmitz et al. 2006) by demonstrating disorder-specificity relative to ADHD. Predominantly, the right (Chambers et al. 2009; Rubia et al. 2003) but also left IFC (Hampshire et al. 2010; Rubia et al. 2011; Rubia et al. 2001; Rubia et al. 2007) are crucial areas mediating response inhibition and ASD patients may have needed to recruit these areas more for comparable task performance. ADHD patients had no underactivation in these areas relative to controls, unlike observed previously (Cubillo et al. 2014; Hart et al. 2013; Rubia 2011; Rubia et al. 1999; Rubia et al. 2005b), but relative to ASD. This, and the lack of a significant difference between controls and ADHD boys in SSRT may be due to long-term effects of stimulant medication, which has been associated with more normal prefrontal and striatal function (Hart et al. 2012; Hart et al. 2013; Rubia et al. 2014), as 11 of the ADHD boys were on chronic methylphenidate medication, even if taken off medication prior to the scan. It is also interesting to note that the enhanced omission errors which were observed in ADHD patients relative to both control and ASD boys under placebo were correlated with reduced IFC-striatal activation under placebo. It has been suggested that omission errors are indicative of an alternative strategy that ADHD boys may employ to decrease their number of failed inhibitions (Tannock et al. 1989). Therefore, although there was no behavioural evidence for poor inhibitory performance using the inhibitory measure of the SSRT, there were significant behavioural differences in omission errors in ADHD patients under placebo. The differences correlated with the neurofunctional deficits and were no longer observed under fluoxetine. The disorder-exclusive deficits in ADHD adolescents in the left OFC/basal ganglia relative to controls extend prior findings of OFC-striatal deficits in ADHD (Cubillo et al. 2014; Hart et al. 2013; Rubia 2011; Rubia et al. 1999; Rubia et al. 2005b; Rubia et al. 2014). Our results therefore show disorder-dissociated frontal lobe dysfunctions in ADHD and ASD boys during inhibition relative to controls: with ASD patients showing significantly enhanced bilateral IFC activation and ADHD boys showing significantly reduced OFC/basal ganglia activation, which furthermore correlated with their enhanced omission errors during task performance.

Fluoxetine normalized all disorder-dissociated frontal brain dysfunctions due to opposite modulation effects in both disorders, which were, however, only significant in the ASD group. Fluoxetine significantly reduced bilateral OFC activation in the ASD group but enhanced it in ADHD. Under fluoxetine, ADHD patients also no longer showed enhanced omission errors, which were reduced under placebo and correlated with the OFC deficits. Fluoxetine also had an opposite up/downregulation effect on another key inhibition region, the pre-SMA: this was upregulated in ASD and hence increased relative to controls and ADHD, but downregulated in ADHD and hence reduced relative to controls and ASD. The upregulation effect of fluoxetine on right IFC and left OFC-striatal regions in ADHD extends prior evidence on SE agonist modulation of these areas in healthy adults during inhibitory control (Del-Ben et al. 2005) to the ADHD population. Most intriguing, however, are the consistently opposite reduction effects of fluoxetine on these frontal activations in the ASD group. Opposite activation effects could possibly reflect group differences in baseline SE levels, as evidenced by lower platelet SE levels in ADHD children compared to controls (Spivak et al. 1999), and increased baseline platelet, blood and prefrontal SE levels in 30 % of individuals with ASD relative to controls (Hranilovic et al. 2007; Mulder et al. 2004; Nakamura et al. 2010; Piven et al. 1991). In addition, ASD patients have abnormal 5-HT synthesis (Chugani et al. 1997), 5-HT transporter (Makkonen et al. 2008; Nakamura et al. 2010) and 5-HT_2A_ receptor binding (Murphy et al. 2006). Therefore, fluoxetine may increase the low SE baseline of children with ADHD to normal levels, leading to normalized activation in fronto-striatal areas. Whereas in children with ASD, the increase in SE in an already hyperserotonemic system may activate a negative feedback mechanism via activation of 5-HT_1A_ autoreceptors (Sibley et al. 2007), leading to a decrease in SE and in fronto-striatal activation.

Despite significant modulation effects on brain activation, fluoxetine had no effect on inhibitory performance. However, the enhanced omission errors that were observed in ADHD patients under placebo relative to controls were no longer significant under fluoxetine. Furthermore, it is well documented that brain function is more sensitive to pharmacological manipulations than inhibitory performance (Cubillo et al. 2014; Rubia et al. 2011; Rubia et al. 2005a). In addition, one of the upregulated regions in ASD in a key inhibitory area, the pre-SMA, was correlated with SSRT, suggesting a relationship between activation changes and inhibitory performance.

The strengths of this study are carefully selected and non-comorbid patient groups who had no psychiatric comorbidities and, in the case of the ASD group, were medication-naïve. A limitation of this study is that for ethical and financial reasons, the control group was scanned only once, while patients were scanned twice which could have accounted for the lack of performance differences. The significantly lower IQ in the ADHD compared to the other two groups is another limitation. However, covariance analysis showed that the findings were not affected by IQ. Furthermore, stop task performance has been shown to be independent of IQ (Friedman et al. 2006). Lastly, while the contrast of successful and unsuccessful stop trials controls for the attentional oddball effect of the low frequency of stop stimuli, it may be over-conservative, as during unsuccessful stop trials subjects may also attempt, but not achieve to inhibit.

## Conclusions

To summarise, ADHD and ASD patients showed opposite brain activation abnormalities relative to controls: ADHD boys showed reduced while ASD boys enhanced frontal activation. Importantly, fluoxetine had a disorder-dissociated opposite effect on frontal brain dysfunctions, enhancing frontal activation in ADHD and reducing it in ASD which could suggest differential underlying SE baseline levels in the two disorders.

## Electronic supplementary material

Below is the link to the electronic supplementary material.

ESM 1 (DOCX 214 kb)

## References

[CR1] Alderson R, Rapport M, Kofler M (2007). Attention-deficit/hyperactivity disorder and behavioral inhibition: a meta-analytic review of the stop-signal paradigm. J Abnorm Child Psychol.

[CR2] American Psychiatric Association (2000). Diagnostic and Statistical Manual of Mental Disorders.

[CR3] Anderson IM, McKie S, Elliott R, Williams SR, Deakin JFW (2008). Assessing human 5-HT function in vivo with pharmacoMRI. Neuropharmacology.

[CR4] Barrickman L, Noyes R, Kuperman S, Schumacher E, Verda M (1991). Treatment of ADHD with fluoxetine—a preliminary trial. J Am Acad Child Adolesc Psychiatry.

[CR5] Brammer MJ, Bullmore ET, Simmons A, Williams SCR, Grasby PM, Howard RJ (1997). Generic brain activation mapping in functional magnetic resonance imaging: a nonparametric approach. Magn Reson Imaging.

[CR6] Bridgett DJ, Walker ME (2006). Intellectual functioning in adults with ADHD: a meta-analytic examination of full scale IQ differences between adults with and without ADHD. Psychol Assess.

[CR7] Bullmore E, Brammer M, Rabe-Hesketh S, Curtis V, Morris R, Williams S (1999). Methods for diagnosis and treatment of stimulus-correlated motion in generic brain activation studies using fMRI. Hum Brain Mapp.

[CR8] Bullmore E, Long C, Suckling J, Fadili J, Calvert G, Zelaya F (2001). Colored noise and computational inference in neurophysiological (fMRI) time series analysis: resampling methods in time and wavelet domains. Hum Brain Mapp.

[CR9] Bullmore ET, Suckling J, Overmeyer S, Rabe-Hesketh S, Taylor E, Brammer MJ (1999). Global, voxel, and cluster tests, by theory and permutation, for a difference between two groups of structural MR images of the brain. IEEE Trans Med Imaging.

[CR10] Chamberlain SR, Muller U, Blackwell AD, Clark L, Robbins TW, Sahakian BJ (2006). Neurochemical modulation of response inhibition and probabilistic learning in humans. Science.

[CR11] Chambers CD, Garavan H, Bellgrove MA (2009). Insights into the neural basis of response inhibition from cognitive and clinical neuroscience. Neurosci Biobehav Rev.

[CR12] Chugani DC, Muzik O, Rothermel R, Behen M, Chakraborty P, Mangner T (1997). Altered serotonin synthesis in the dentatothalamocortical pathway in autistic boys. Ann Neurol.

[CR13] Conners CK, Sitarenios G, Parker JDA, Epstein JN (1998). Revision and restandardization of the Conners Teacher Rating Scale (CTRS-R): factor structure, reliability, and criterion validity. J Abnorm Child Psychol.

[CR14] Corbett BA, Constantine LJ, Hendren R, Rocke D, Ozonoff S (2009). Examining executive functioning in children with autism spectrum disorder, attention deficit hyperactivity disorder and typical development. Psychiatry Res.

[CR15] Cortese S, Kelly C, Chabernaud C, Proal E, Di Martino A, Milham MP (2012). Toward systems neuroscience of ADHD: a meta-analysis of 55 fMRI studies. Am J Psychiatry.

[CR16] Cubillo A, Halari R, Smith A, Taylor E, Rubia K (2012). A review of fronto-striatal and fronto-cortical brain abnormalities in children and adults with attention deficit hyperactivity disorder (ADHD) and new evidence for dysfunction in adults with ADHD during motivation and attention. Cortex.

[CR17] Cubillo A, Smith A, Barrett N, Giampetro V, Brammer M, Simmons A et al. (2014) Shared and drug specific effects of atomoxetine and methylphenidate on inhibitory brain dysfunction in medication-naive ADHD boys. Cerebal Cortex 24(1):174–185 10.1093/cercor/bhs296 PMC3862268 23048018

[CR18] Del-Ben CM, Deakin JFW, McKie S, Delvai NA, Williams SR, Elliott R (2005). The effect of citalopram pretreatment on neuronal responses to neuropsychological tasks in normal volunteers: an fMRI study. Neuropsychopharmacology.

[CR19] DeLong GR, Ritch CR, Burch S (2002). Fluoxetine response in children with autistic spectrum disorders: correlation with familial major affective disorder and intellectual achievement. Dev Med Child Neurol.

[CR20] DeLong GR, Teague LA, Kamran MM (1998). Effects of fluoxetine treatment in young children with idiopathic autism. Dev Med Child Neurol.

[CR21] Dennis M, Francis DJ, Cirino PT, Schachar R, Barnes MA, Fletcher JM (2009). Why IQ is not a covariate in cognitive studies of neurodevelopmental disorders. J Int Neuropsychol Soc.

[CR22] Friedman NP, Miyake A, Corley RP, Young SE, DeFries JC, Hewitt JK (2006). Not all executive functions are related to intelligence. Psychol Sci.

[CR23] Gammon GD, Brown TE (1993). Fluoxetine and methylphenidate in combination for treatment of attention deficit disorder and comorbid depressive disorder. J Child Adolesc Psychopharmacol.

[CR24] Geurts HM, Vertie S, Oosterlaan J, Roeyers H, Sergeant JA (2004). How specific are executive functioning deficits in attention deficit hyperactivity disorder and autism?. J Child Psychol Psychiatry.

[CR25] Goldberg DP, Murray R (2006). The Maudsley Handbook of Practical Psychiatry.

[CR26] Goodman R, Scott S (1999). Comparing the strengths and difficulties questionnaire and the child behavior checklist: is small beautiful?. J Abnorm Child Psychol.

[CR27] Hampshire A, Chamberlain SR, Monti MM, Duncan J, Owen AM (2010). The role of the right inferior frontal gyrus: inhibition and attentional control. Neuroimage.

[CR28] Hart H, Radua J, Mataix-Cols D, Rubia K (2012). Meta-analysis of fMRI studies of timing in attention-deficit hyperactivity disorder (ADHD). Neurosci Biobehav Rev.

[CR29] Hart H, Radua J, Nakao T, Mataix-Cols D, Rubia K (2013). Meta-analysis of functional magnetic resonance imaging studies of inhibition and attention in attention-deficit/hyperactivity disorder: exploring task-specific, stimulant medication, and age effects. JAMA Psychiatry.

[CR30] Hollander E, Phillips A, Chaplin W, Zagursky K, Novotny S, Wasserman S (2005). A placebo controlled crossover trial of liquid fluoxetine on repetitive behaviors in childhood and adolescent autism. Neuropsychopharmacology.

[CR31] Hranilovic D, Bujas-Petkovic Z, Vragovic R, Vuk T, Hock K, Jernej B (2007). Hyperserotonemia in adults with autistic disorder. J Autism Dev Disord.

[CR32] Kana RK, Keller TA, Minshew NJ, Just MA (2007). Inhibitory control in high-functioning autism: decreased activation and underconnectivity in inhibition networks. Biol Psychiatry.

[CR33] King BH, Hollander E, Sikich L, McCracken JT, Scahill L, Bregman JD (2009). Lack of efficacy of citalopram in children with autism spectrum disorders and high levels of repetitive behavior citalopram ineffective in children with autism. Arch Gen Psychiatry.

[CR34] Lamar M, Craig M, Daly EM, Cutter WJ, Tang C, Brammer M et al. (2012) Acute tryptophan depletion promotes an anterior-to-posterior fMRI activation shift during task switching in older adults. Hum Brain Mapp In Press 10.1002/hbm.22187 PMC6868962 23281064

[CR35] Lamar M, Cutter WJ, Rubia K, Brammer M, Daly EM, Craig MC (2009). 5-HT, prefrontal function and aging: fMRI of inhibition and acute tryptophan depletion. Neurobiol Aging.

[CR36] Langen M, Durston S, Kas MJH, van Engeland H, Staal WG (2011). The neurobiology of repetitive behavior: … and men. Neurosci Biobehav Rev.

[CR37] Lipsyzc J, Schachar R (2010). Inhibitory control and psychopathology: a meta-analysis of studies using the stop signal task. J Int Neuropsychol Soc.

[CR38] Logan GD, Schachar RJ, Tannock R (1997). Impulsivity and inhibitory control. Psychol Sci.

[CR39] Lord C, Risi S, Lambrecht L, Cook EH, Leventhal BL, DiLavore PC (2000). The autism diagnostic observation schedule generic: a standard measure of social and communication deficits associated with the spectrum of autism. J Autism Dev Disord.

[CR40] Lord C, Rutter M, Couteur A (1994). Autism diagnostic interview-revised: a revised version of a diagnostic interview for caregivers of individuals with possible pervasive developmental disorders. J Autism Dev Disord.

[CR41] Makkonen I, Riikonen R, Kokki H, Airaksinen MM, Kuikka JT (2008). Serotonin and dopamine transporter binding in children with autism determined by SPECT. Dev Med Child Neurol.

[CR42] Matthews JN, Altman DG (1996). Statistics notes. Interaction 2: compare effect sizes not *P* values. BMJ.

[CR43] Mulder EJ, Anderson GM, Kema IP, de Bildt A, van Lang NDJ, den Boer JA (2004). Platelet serotonin levels in pervasive developmental disorders and mental retardation: diagnostic group differences, within-group distribution, and behavioral correlates. J Am Acad Child Adolesc Psychiatry.

[CR44] Murphy DGM, Daly E, Schmitz N, Toal F, Murphy K, Curran S (2006). Cortical serotonin 5-HT2A receptor binding and social communication in adults with Asperger’s syndrome: an in vivo SPECT study. Am J Psychiatry.

[CR45] Nakamura K, Sekine Y, Ouchi Y, Tsujii M, Yoshikawa E, Futatsubashi M (2010). Brain serotonin and dopamine transporter bindings in adults with high-functioning autism. Arch Gen Psychiatry.

[CR46] Oades RD (2007). Role of the serotonin system in ADHD: treatment implications. Expert Rev Neurother.

[CR47] Oldfield RC (1971). The assessment and analysis of handedness: the Edinburgh inventory. Neuropsychologia.

[CR48] Ozonoff S, Strayer DL (1997). Inhibitory function in nonretarded children with autism. J Autism Dev Disord.

[CR49] Pauls AM, O’Daly OG, Rubia K, Riedel WJ, Williams SC, Mehta MA (2012). Methylphenidate effects on prefrontal functioning during attentional-capture and response inhibition. Biol Psychiatry.

[CR50] Pauls AM, O’Daly OG, Rubia K, Riedel WJ, Williams SC, Mehta MA (2012). Methylphenidate effects on prefrontal functioning during attentional-capture and response inhibition. Biol Psychiatry.

[CR51] Piven J, Tsai G, Nehme E, Coyle JT, Chase GA, Folstein SE (1991). Platelet serotonin, a possible marker for familial autism. J Autism Dev Disord.

[CR52] Quintana H, Butterbaugh GJ, Purnell W, Layman AK (2007). Fluoxetine monotherapy in attention-deficit/hyperactivity disorder and comorbid non-bipolar mood disorders in children and adolescents. Child Psychiatry Hum Dev.

[CR53] Raymaekers R, Antrop I, van der Meere JJ, Wiersema JR, Roeyers H (2007). HFA and ADHD: a direct comparison on state regulation and response inhibition. J Clin Exp Neuropsychol.

[CR54] Robbins TW, Crockett MJ, Christian PM, Barry LJ (2010) Role of central serotonin in impulsivity and compulsivity: comparative studies in experimental animals and humans. Handbook of Behavioral Neuroscience. Elsevier. Volume 21, pp 415–427

[CR55] Robinson S, Goddard L, Dritschel B, Wisley M, Howlin P (2009). Executive functions in children with autism spectrum disorders. Brain Cogn.

[CR56] Rommelse NNJ, Franke B, Geurts HM, Hartman CA, Buitelaar JK (2010). Shared heritability of attention-deficit/hyperactivity disorder and autism spectrum disorder. Eur Child Adolesc Psychiatry.

[CR57] Rommelse NNJ, Geurts HM, Franke B, Buitelaar JK, Hartman CA (2011). A review on cognitive and brain endophenotypes that may be common in autism spectrum disorder and attention-deficit/hyperactivity disorder and facilitate the search for pleiotropic genes. Neurosci Biobehav Rev.

[CR58] Rubia K (2011). “Cool” inferior frontostriatal dysfunction in attention-deficit/hyperactivity disorder versus “hot” ventromedial orbitofrontal-limbic dysfunction in conduct disorder: a review. Biol Psychiatry.

[CR59] Rubia K, Alegria A, Brinson H (2014) Imaging the ADHD brain: disorder-specificity, medication effects and clinical translation. Expert Rev Neurother 14(5):519–538 10.1586/14737175.2014.907526 24738703

[CR60] Rubia K, Halari R, Mohammad A-M, Taylor E, Brammer M (2011). Methylphenidate normalizes frontocingulate underactivation during error processing in attention-deficit/hyperactivity disorder. Biol Psychiatry.

[CR61] Rubia K, Lee F, Cleare AJ, Tunstall N, Fu CHY, Brammer M (2005). Tryptophan depletion reduces right inferior prefrontal activation during response inhibition in fast, event-related fMRI. Psychopharmacology (Berl).

[CR62] Rubia K, Overmeyer S, Taylor E, Brammer M, Williams SCR, Simmons A (1999). Hypofrontality in attention deficit hyperactivity disorder during higher-order motor control: a study with functional MRI. Am J Psychiatry.

[CR63] Rubia K, Russell T, Overmeyer S, Brammer MJ, Bullmore ET, Sharma T (2001). Mapping motor inhibition: conjunctive brain activations across different versions of go/no-go and stop tasks. Neuroimage.

[CR64] Rubia K, Smith AB, Brammer MJ, Taylor E (2003). Right inferior prefrontal cortex mediates response inhibition while mesial prefrontal cortex is responsible for error detection. Neuroimage.

[CR65] Rubia K, Smith AB, Brammer MJ, Toone B, Taylor E (2005). Abnormal brain activation during inhibition and error detection in medication-naive adolescents with ADHD. Am J Psychiatry.

[CR66] Rubia K, Smith AB, Taylor E, Brammer M (2007). Linear age-correlated functional development of right inferior fronto-striato-cerebellar networks during response inhibition and anterior cingulate during error-related processes. Hum Brain Mapp.

[CR67] Rutter M, Bailey A, Lord C (2003) The social communication questionnaire: manual western psychological services: Los Angeles

[CR68] Schmitz N, Rubia K, Daly E, Smith A, Williams S, Murphy DGM (2006). Neural correlates of executive function in autistic spectrum disorders. Biol Psychiatry.

[CR69] Sibley D, Hanin I, Kuhar M, Skolnick P (2007). Handbook of Contemporary Neuropharmacology Vol. 1.

[CR70] Simonoff E, Pickles A, Charman T, Chandler S, Loucas T, Baird G (2008). Psychiatric disorders in children with autism spectrum disorders: prevalence, comorbidity, and associated factors in a population-derived sample. J Am Acad Child Adolesc Psychiatry.

[CR71] Sinzig J, Lehmkuhl G (2007). What do we know about the serotonergic genetic heterogeneity in attention-deficit/hyperactivity and autistic disorders?. Psychopathology.

[CR72] Spivak B, Vered Y, Yoran-Hegesh R, Averbuch E, Mester R, Graf E (1999). Circulatory levels of catecholamines, serotonin and lipids in attention deficit hyperactivity disorder. Acta Psychiatr Scand.

[CR73] Tannock R, Schachar R, Carr R, Chajczyk D, Logan G (1989). Effects of methylphenidate on inhibitory control in hyperactive children. J Abnorm Child Psychol.

[CR74] Thirion B, Pinel P, Meriaux S, Roche A, Dehaene S, Poline JB (2007). Analysis of a large fMRI cohort: statistical and methodological issues for group analyses. Neuroimage.

[CR75] van der Meer JMJ, Oerlemans AM, van Steijn DJ, Lappenschaar MGA, de Sonneville LMJ, Buitelaar JK (2012). Are autism spectrum disorder and attention-deficit/hyperactivity disorder different manifestations of one overarching disorder? Cognitive and symptom evidence from a clinical and population-based sample. J Am Acad Child Adolesc Psychiatry.

[CR76] Wechsler D (1999). Wechsler Abbreviated Scale of Intelligence.

[CR77] Wong DT, Bymaster FP, Engleman EA (1995). Prozac (fluoxetine, Lilly 110140), the first selective serotonin uptake inhibitor and an antidepressant drug: 20 years since its first publication. Life Sci.

[CR78] World Health Organisation (1994). ICD-10 classification of mental and behavioural disorders: clinical descriptions and diagnostic guidelines.

[CR79] Zafeiriou DI, Ververi A, Vargiami E (2009). The serotonergic system: its role in pathogenesis and early developmental treatment of autism. Curr Neuropharmacol.

