# Supplementary material for: Inverse fluoxetine effects on inhibitory brain activation in non-comorbid boys with ADHD and with ASD
Source: Psychopharmacology (Berl). 2014 Dec 24;232(12):2071–82. doi: 10.1007/s00213-014-3837-2 (PMC4432080; doi:10.1007/s00213-014-3837-2)
Supplement: Supplementary file 1 — (DOCX 214 kb) [file 213_2014_3837_MOESM1_ESM.docx]

**SUPPLEMENTARY MATERIAL**

**Methods**

**Recruitment:**

Thirty - two ADHD boys were recruited in total, however 7 boys dropped out of the study due to their dislike of the MRI scanner, 3 were excluded due to co-morbidities (despite the fact we explicitly aimed to recruit only non-comorbid cases), 1 boy did not reach the diagnostic criteria for the combined subtype of ADHD, 1 boy was excluded due to poor task performance and 2 were excluded due to high levels of motion.

Forty – four ASD boys were recruited in total. Of these, 7 boys dropped out of the study due to their dislike of the MRI scanner, 14 were excluded due to co-morbidities, 1 was excluded due to neurological abnormalities, 2 were excluded due to SSRI use and 1 was excluded due to poor task performance.

Thirty – four controls were recruited in total however 9 were excluded due to high scores on the SDQ and CPRS.

Fluoxetine was titrated for age and weight in the following manner: boys between 10-13 years and < 30kg received 8mg, those > 30kg received 10mg. Boys between 14-17 years and < 30kg received 10mg, those > 30kg received 15mg.

No side effects were reported for any of the patients with the exception of a nose-bleed in one of the patients. It was, however, not clear whether it was related to the medication or a random effect.

**fMRI Data Analysis Methods**

*Individual Analysis* - fMRI data were first processed to minimise motion related artefacts (Bullmore *et al.,* 1999a). A 3D volume consisting of the average intensity at each voxel over the whole experiment was calculated and used as a template. The 3D image volume at each time point was then realigned to this template by computing the combination of rotations (around the x y and z axes) and translations (in x y and z) that maximised the correlation between the image intensities of the volume in question and the template (rigid body registration). Following realignment, data were then smoothed using a Gaussian filter (FWHM, 7.2mm) to improve the signal to noise characteristics of the images. After motion correction, global detrending and spin-excitation history correction, time series analysis for each subject was based on a wavelet-based data resampling method for functional MRI data (Bullmore *et al.,* 1999b. 2001). At the individual subject level, a standard general linear modelling approach was used to obtain estimates of the response size (beta) to the stop signal task conditions (successful and failed stop trials) against an implicit baseline (go trials) and then again for the higher level contrast of successful-go trials minus unsuccessful-go trials. Briefly, we first convolved the main experimental condition (successful and failed Stop trials, each separately contrasted with Go trials) and the higher level contrast (successful Stop-go trials minus unsuccessful Stop-go trials) with two Poisson model functions (peaking at 4s and 8s) after motion correction, global detrending and spin-excitation history correction. We then calculated the weighted sum of these two convolutions that gave the best fit (least-squares) to the time series at each voxel. A goodness-of-fit statistic (the SSQ-ratio) was then computed at each voxel consisting of the ratio of the sum of squares of deviations from the mean intensity value due to the model (fitted time series) divided by the sum of squares due to the residuals (original time series minus model time series). The appropriate null distribution for assessing significance of any given SSQ-ratio was established using a wavelet-based data re-sampling method (Bullmore *et al.,* 2001) and applying the model-fitting process to the re-sampled data. This process was repeated 20 times at each voxel and the data combined over all voxels, resulting in 20 null parametric maps of SSQ-ratio for each subject, which were combined to give the overall null distribution of SSQ-ratio. The same permutation strategy was applied at each voxel to preserve spatial correlation structure in the data. Activated voxels, at a <1 level of type I error, were identified through the appropriate critical value of the SSQ-ratio from the null distribution (Bullmore *et al.,* 1999b, Brammer *et al.,* 1997). Individual SSQ-ratio maps were then transformed into standard space, first by rigid body transformation of the fMRI data into a high-resolution inversion recovery image of the same subject, and then by affine transformation onto a Talairach template (Talairach & Tounrnaux1988).

*Group Analysis* - A group activation map was produced for the experimental condition (Successful Stop—Unsuccessful Stop) by calculating the median observed SSQ-ratio over all subjects at each voxel in standard space and testing them against the null distribution of median SSQ-ratios computed from the identically transformed wavelet re-sampled data (Bullmore *et al*., 2001, Brammer *et al*., 1997). The voxel-level threshold was first set to 0.05 to give maximum sensitivity and to avoid type II errors. Next, a cluster-level threshold was computed for the resulting 3D voxel clusters. The necessary combination of voxel and cluster level thresholds was not assumed from theory but rather was determined by direct permutation for each data set, giving excellent Type II error control (Bullmore *et al*., 1999b). Cluster mass rather than a cluster extent threshold was used, to minimise discrimination against possible small, strongly responding foci of activation (Bullmore *et al.,* 1999b). In all group activation analyses, less than one false positive activation locus was expected for p<0.05 at voxel level and p<0.01 at cluster level.

**Results**

**Group differences in clinical questionnaire measures**

Multivariate ANOVA showed a significant group effect for all SDQ measures (F(df =10,112)=30 p < 0.0001). Post-hoc analyses are shown in the supplement. Post-hoc analyses showed that controls scored significantly better on all subscales compared to patients (p < 0.001). ADHD boys scored significantly higher than ASD boys on the conduct and hyperactive/inattentive subscales of the SDQ (p < 0.0001) while ASD boys scored significantly worse than ADHD boys on the peer relations and prosocial subscales (p < 0.05) and significantly higher on the SCQ than ADHD and controls participants, while ADHD participants scored significantly higher than controls (F (df =2,55) = 152, p < 0.0001). ADHD boys scored higher on the CPRS than ASD and controls and ASD participants scored higher than controls (F (df =2,56) = 192, p< 0.0001) (Table1).

**Group activations within controls and ADHD patients under each drug condition for the two contrasts of the Stop task**

**Successful Inhibition- Failed Inhibition**

**Controls:** Controls activated a typical inhibition network consisting of right inferior and middle frontal cortex, right caudate, bilateral putamen/globus pallidus, right middle/superior temporal lobe (STL), right inferior parietal lobe, occipital lobe and left cerebellum.

**ADHD:** While on placebo, the ADHD group activated bilateral occipital lobe. While on Fluoxetine, they activated right middle and superior frontal cortex, bilateral precentral and postcentral gyri, right caudate, putamen, insula and thalamus, right temporal lobe, bilateral parietal lobe, posterior cingulate cortex (PCC), bilateral occipital lobe and cerebellum/midbrain.

**ASD:** While on placebo, the ASD group activated bilateral middle and superior frontal cortex, right IFC, right caudate, putamen, thalamus and precuneus, bilateral occipital lobe and cerebellum. While on Fluoxetine, the ASD group activated right precentral and postcentral gyri, right parietal lobe, right middle temporal, bilateral occipital lobe, precuneus and left cerebellum.

(see Supplementary Figure 1 A,B,C)

**Failed inhibition - Successful inhibition**

**Controls:** Controls activated a single cluster in right precentral and postcentral gyri.

**ADHD:** While on placebo, the ADHD group activated a single cluster in left precentral and postcentral gyri reaching into parietal lobe. While on Fluoxetine, the ADHD group activated medial prefrontal cortex (mPFC)/pre-SMA reaching into left superior frontal cortex and left inferior parietal lobe.

**ASD:**  While on placebo, the ASD group activated mPFC, including ACC, PCC and a left hemispheric network consisting of IFC, post insula, putamen and premotor and postcentral gyri. While on Fluoxetine, they activated ACC and left postcentral gyrus.

(see Supplementary Figure 2 A,B,C).

**
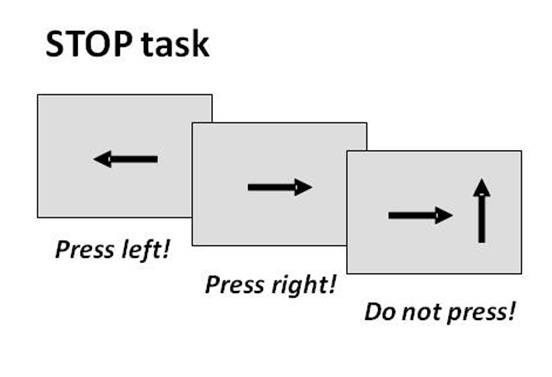
**

**Supplementary Figure 1. Schematic presentation of the tracking Stop task.** Subjects have to respond to go arrows that point either right or left with a right/left button response. In 20% of trials, the go-signals are followed (about 250ms later) by stop signals and subjects had to inhibit their motor responses. A tracking algorithm changes the time interval between go-signals and stop-signals according to each subject’s performance on previous trials (average percentage of inhibition over previous stop trials, recalculated after each stop trial), resulting in 50% successful and 50% unsuccessful inhibition trials.


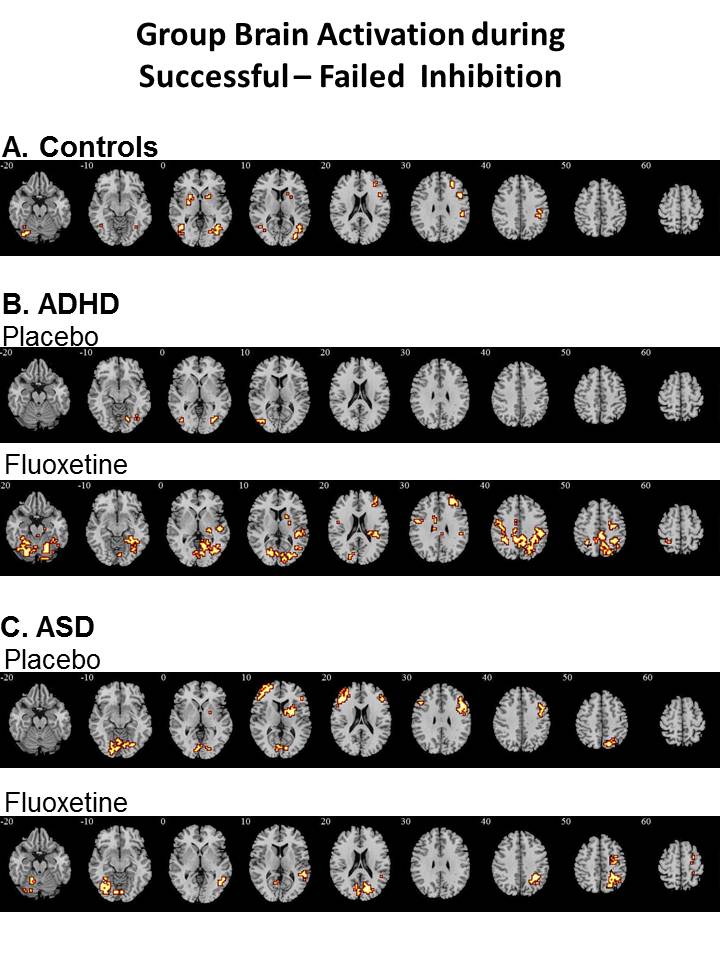


**Supplementary Figure S2. Within-group activation for A. Healthy controls, B. Adolescents with ADHD under either placebo or Fluoxetine and C. Adolescents with ASD under either placebo or Fluoxetine for the contrast of successful inhibition – failed inhibition at p < 0.05 for voxel and p < 0.01 for cluster-level, leading to less than 1 error clusters**. Axial sections showing within-group brain activation for healthy control boys, boys with ADHD under either placebo or Fluoxetine and boys with ASD under either placebo or Fluoxetine for the contrast of successful – failed inhibition. Talairach z co-ordinates are indicated for slice distance (in mm) from the intercommissural line. The right side corresponds to the right side of the image.


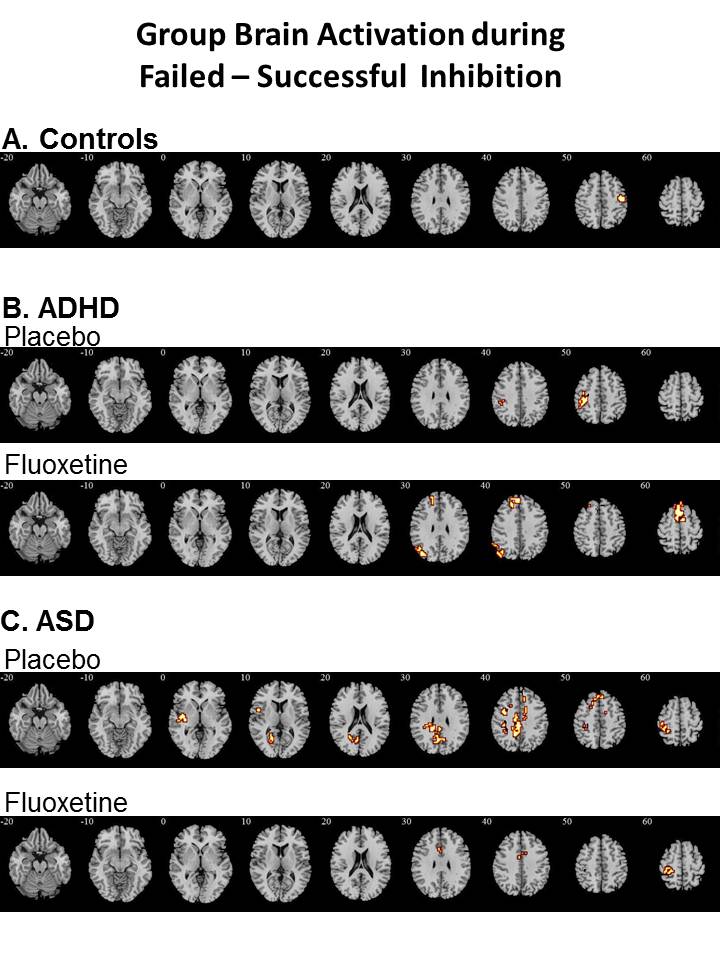


**Supplementary Figure S3**. **Within-group activation for A. Healthy controls, B. Adolescents with ADHD under either placebo or Fluoxetine and C. Adolescents with ASD under either placebo or Fluoxetine for the contrast of failed inhibition– successful inhibition at p < 0.05 for voxel and p < 0.01 for cluster-level, leading to less than 1 error clusters.** Axial sections showing within-group brain activation for healthy control boys, boys with ADHD under either placebo or Fluoxetine and boys with ASD under either placebo or Fluoxetine for the contrast of failed – successful inhibition. Talairach z-co-ordinates are indicated for slice distance (in mm) from the intercommissural line. The right side corresponds to the right side of the image. The right side corresponds to the right side of the image.

**References**

Bullmore E, Brammer M, Rabe-Hesketh S, Curtis V, et al. (1999a): Methods for diagnosis and treatment of stimulus-correlated motion in generic brain activation studies using fMRI. Hum Brain Mapp. 7:38-48.

Bullmore E, Long C, Suckling J, Fadili J, Calvert G, Zelaya F, et al. (2001): Colored noise and computational inference in neurophysiological (fMRI) time series analysis: resampling methods in time and wavelet domains. Hum Brain Mapp. 12:61-78.

Bullmore ET, Suckling J, Overmeyer S, Rabe-Hesketh S, et al. (1999b): Global, voxel, and cluster tests, by theory and permutation, for a difference between two groups of structural MR images of the brain. IEEE Trans Med Imaging 18:32-42.

Brammer MJ, Bullmore ET, Simmons A, Williams SC, et al. (1997): Generic brain activation mapping in functional magnetic resonance imaging: a nonparametric approach. Magnetic Resonance Imaging 15:763-770.

Talairach J, Tournoux P (1988): Co-planar stereotaxic atlas of the brain. New York: Thieme.
